# Judith Campisi (1950-2024) - A Pioneering Geroscientist in Senescence and SASP

**DOI:** 10.14336/AD.2024.0123

**Published:** 2024-04-01

**Authors:** Kunlin Jin

**Affiliations:** Department of Pharmacology & Neuroscience, University of North Texas Health Science Center, Fort Worth, TX 76107, USA.

Judith Campisi, a pioneering geroscientist, passed away on January 19, 2024, at the age of 74. A leader in cellular senescence and the Senescence-Associated Secretory Phenotype (SASP), Campisi's seminal work fundamentally shifted our understanding of these processes from a cancer-centric view to recognizing their intricate involvement in aging. Her discovery that senescent cells, which cease dividing but do not die, secrete factors influencing their surroundings was pivotal. These factors, including inflammatory cytokines, growth factors, and proteases, have dual roles, being both beneficial and detrimental.

As an esteemed member of the National Academy of Sciences and a fellow of the American Association for the Advancement of Science, Campisi's influence extended beyond her laboratory. Her contributions to the SENS Research Foundation Advisory Board and the Lifeboat Foundation were notable. She also held editorial roles, serving as co-Editor-in-Chief of *Aging* and Editor in *Aging and Disease*.

Campisi's academic journey began with a B.A. in chemistry (1974) and a Ph.D. in biochemistry (1979) from the State University of New York at Stony Brook. Her postdoctoral training at the Dana-Farber Cancer Institute and Harvard Medical School (1982) focused on cell cycle regulation. During her tenure at Boston University Medical School (1983-1991), she explored cellular senescence's role in cancer suppression, laying the groundwork for her later research linking senescence and aging. Before joining the Buck Institute for Research on Aging in 2002, she was a Senior Scientist at the Lawrence Berkeley National Laboratory.

Her work elucidated the paradoxical nature of senescence and cancer: while senescence prevents damaged cells from proliferating, thus acting as a cancer barrier, SASP can foster an environment that promotes cancer in aged tissues.

Beyond her scientific acumen, Campisi was a mentor and advocate for interdisciplinary aging research. Her approach wasn't just about the passage of time; she viewed aging as a complex biological phenomenon with profound health and disease implications. My tenure as a Research Professor at the Buck Institute from 1999 to 2011 overlapped with Campisi’s, providing me the opportunity to witness her mentorship and collaborative spirit firsthand. Her support for my work continued even after I relocated to Texas, particularly through the journal *Aging and Disease* and within the *International Society on Aging and Disease* (ISOAD) (www.isoad.org). Her unwavering commitment to advancing our field and nurturing future scientists was nothing short of inspiring.

Reflecting on Judith Campisi's career, her contributions have been instrumental in shaping gerontology and biogerontology. Her deep insights into cellular senescence have not only enriched our understanding of aging but also opened avenues for therapeutic strategies aimed at enhancing healthspan and life quality in the elderly. In her memory, I aspire to uphold her ideals and continue her legacy, fostering the same spirit of inquiry and collaboration that she championed throughout her remarkable career.

